# Effect of varicocele surgical repair on antisperm antibody positivity rate in patients with clinical varicocele: a systematic review and meta-analysis

**DOI:** 10.3389/fendo.2026.1925219

**Published:** 2026-08-12

**Authors:** Shengnan Li, Tengfei Chen, Boxian Gao, Chongfu Zhong

**Affiliations:** 1 First Clinical Medical College of Shandong University of Traditional Chinese Medicine, Jinan, China; 2 Department of Urology, Affiliated Hospital of Shandong University of Traditional Chinese Medicine, Jinan, China; 3 Andrology Department, Affiliated Hospital of Shandong University of Traditional Chinese Medicine, Jinan, China

**Keywords:** antisperm antibody, male infertility, meta-analysis, varicocele, varicocele repair

## Abstract

**Background:**

Varicocele is a common correctable cause of male infertility, and antisperm antibodies (ASA) have been detected at varying rates in affected men. However, the effect of varicocele repair on ASA positivity remains controversial, and no meta-analysis has specifically quantified this outcome in men with clinical varicocele.

**Methods:**

We systematically searched PubMed, Web of Science, Embase, Cochrane Library, CNKI, VIP, Wanfang, and SinoMed up to April 30, 2026. Before-after studies reporting ASA positivity rates or ASA levels in men with clinical varicocele were included. Pooled risk ratios (RRs) with 95% confidence intervals (CIs) were calculated using a random-effects model. Subgroup analyses were performed by surgical technique. Sensitivity analyses and publication bias assessment were conducted.

**Results:**

Ten studies (13 comparisons, 722 patients) were included in the RR analysis. Varicocele repair was associated with a lower ASA positivity rate compared with preoperative levels (RR = 0.42; 95% CI: 0.35-0.52; P < 0.00001). Subgroup analysis according to surgical technique showed lower postoperative ASA positivity rates in the microsurgical, laparoscopic, and high ligation groups, with the lowest pooled RR observed in the microsurgical subgroup (RR = 0.32). A significant subgroup difference was observed (*P* = 0.01). Sensitivity analyses excluding studies with back-calculated data or a 6-month follow-up gave pooled RRs of 0.43 and 0.40, respectively, consistent with the primary analysis. Three studies reporting continuous ASA levels were not pooled because of extreme heterogeneity (I^2^ = 98%) and were described narratively.

**Conclusion:**

Varicocele repair was associated with reduced ASA positivity among men with clinical varicocele, with the lowest pooled RR observed in the microsurgical subgroup. The results are robust across sensitivity analyses. These findings suggest that varicocele repair may influence immune-related alterations reflected by ASA positivity; however, the clinical utility of ASA testing for surgical decision-making remains uncertain.

## 1 Introduction

Varicocele (VC) is characterized by abnormal dilation and tortuosity of the pampiniform plexus within the spermatic cord and represents one of the most common correctable causes of male infertility (1). VC affects approximately 15% of adult men in the general population, with a higher prevalence of up to 35% among men with infertility (2). Although the mechanisms underlying VC-associated reproductive impairment are complex, accumulating evidence suggests that impaired spermatogenesis may result from multiple factors, including increased testicular temperature, oxidative stress, metabolic disturbances, and hormonal alterations (3). These pathological changes can lead to abnormal semen parameters, typically manifested as oligoasthenoteratozoospermia, characterized by reduced sperm concentration, impaired motility, and abnormal morphology, thereby compromising.

male reproductive potential (4).

Male infertility is a multifactorial disorder, and the presence of VC does not necessarily represent the sole cause of impaired fertility. Patients with VC may also present with additional contributing factors, including endocrine abnormalities, genetic defects (such as Y-chromosome microdeletions and chromosomal abnormalities), obstructive disorders, infections, and environmental or lifestyle-related factors (4). Therefore, identifying and addressing potentially reversible causes remains essential in the clinical management of infertile men. Varicocele repair, which has been associated with improvements in semen parameters and pregnancy.

outcomes, remains the primary treatment strategy for appropriately selected patients (5, 6).

In addition to these established mechanisms, immunological alterations may also contribute to VC-associated infertility (7). Antisperm antibodies (ASA) are immunoglobulins that recognize sperm surface antigens and may impair sperm motility, disrupt the acrosome reaction, and reduce fertilization capacity (8, 9). ASA positivity is uncommon in the general male population, with reported rates of approximately 0–2%, but may increase to nearly 28% among patients with VC (10, 11). Consistently, previous studies have demonstrated higher levels of sperm-bound immunoglobulins in infertile men with VC compared with those without VC (12, 13). These findings suggest that VC may promote ASA production through disruption of the blood–testis barrier (BTB), which increases exposure of sperm antigens to the immune system and potentially initiates immune responses (14).

However, the clinical relevance of ASA in VC-associated infertility remains unclear. ASA positivity is not observed in all men with VC, and many affected individuals maintain normal antibody levels. Moreover, ASA can also be detected in infertile men without VC, suggesting that ASA is not specific to VC and may represent only one of several contributing mechanisms underlying male infertility. Therefore, although ASA may reflect VC-related immune disturbances, its role as an independent determinant of impaired fertility remains uncertain.

From a biological perspective, varicocele repair may influence ASA status by improving the testicular microenvironment, potentially restoring blood–testis barrier (BTB) function, and reducing abnormal exposure of sperm antigens to the immune system. However, clinical evidence regarding changes in ASA after varicocele repair remains inconsistent. Some studies have reported decreased postoperative ASA levels (15), whereas others have observed no significant changes or even increased levels (16, 17). These discrepancies may be related to variations in surgical techniques, follow-up duration, ASA detection methods, and patient characteristics, as well as the potential influence of surgery-related inflammation during the early postoperative period. Previous meta-analyses evaluating the outcomes of varicocele repair have primarily focused on conventional semen parameters and sperm DNA fragmentation (18, 19). However, whether varicocele repair affects ASA positivity has not been systematically quantified, and the potential differences among surgical techniques remain unclear. Therefore, a comprehensive evaluation of changes in ASA positivity after varicocele repair is needed to better understand the immunological effects of surgical treatment in patients with clinical VC. To address these knowledge gaps, we conducted a systematic review and meta-analysis of studies evaluating preoperative and postoperative ASA outcomes in patients with clinical VC undergoing varicocele repair. The primary objective was to assess the association between varicocele repair and changes in ASA positivity. Secondary objectives were to explore whether changes in ASA positivity differed among surgical techniques, including microsurgical, laparoscopic, and high-ligation approaches, and to evaluate the robustness of the findings through sensitivity analyses and assessment of publication bias. This study was registered prospectively with the INPLASY platform (registration number: INPLASY202650048; Additional file 1).

## 2 Materials and methods

### 2.1 Search strategy

A systematic literature search was conducted in PubMed, Web of Science, Embase, Cochrane Library, CNKI, VIP, SinoMed, and Wanfang from database inception to April 30, 2026. The search strategy combined controlled vocabulary terms (e.g., Medical Subject Headings) with free-text terms related to varicocele and antisperm antibodies. The following terms were used individually or in combination: “varicocele,” “varicocelectomy,” “varicocele repair,” “antisperm antibody,” “anti-sperm antibody,” “ASA,” “sperm antibody,” and “immunological infertility.” No restrictions were applied regarding language or publication type. Additionally, the reference lists of all included studies were manually screened to identify potentially eligible articles. The detailed PubMed search strategy is presented in **Table 1**, and the complete search strategies for all databases are provided in **Supplementary Material 2**.

**Table 1:** Search strategy for PubMed.

#	Search
#1	“varicocele”[MeSH Terms] OR “varicocele”[Title/Abstract] OR “varicoceles”[Title/Abstract] OR “varicocelectomy”[Title/Abstract] OR “internal spermatic vein”[Title/Abstract]
#2	“varicocelectomy”[MeSH Terms] OR “varicocele repair”[Title/Abstract] OR “surgical repair”[Title/Abstract] OR “high ligation”[Title/Abstract] OR “microsurgical varicocelectomy”[Title/Abstract] OR “laparoscopic varicocelectomy”[Title/Abstract] OR “varicocele ligation”[Title/Abstract]
#3	“antisperm antibodies”[MeSH Terms] OR “antisperm antibody”[Title/Abstract] OR “anti-sperm antibody”[Title/Abstract] OR “ASA”[Title/Abstract] OR “sperm antibody”[Title/Abstract] OR “sperm antibodies”[Title/Abstract] OR “sperm agglutinating antibody”[Title/Abstract] OR “sperm surface antibody”[Title/Abstract] OR “immunological infertility”[Title/Abstract]
#4	#1 AND #2 AND #3

The PubMed search was performed from database inception to April 30, 2026, using a combination of Medical Subject Headings (MeSH) and free-text terms in the title or abstract fields. The search strategy was structured around three core concepts: varicocele and related conditions, surgical intervention, and antisperm antibodies. These concepts were combined using the Boolean operator “AND,” while synonyms within each concept were combined with “OR.” No language or publication type restrictions were applied. The reference lists of included studies were also manually screened to identify additional relevant articles.

### 2.2 Inclusion criteria

Studies were eligible for inclusion if they met the following criteria: (1) Study design: before-and-after (pre-post) studies, prospective or retrospective cohort studies, or randomized controlled trials; (2) Population: adult male patients (≥18 years) with clinically diagnosed varicocele, regardless of fertility status; (3) Intervention: patients who underwent surgical varicocele repair, including any surgical technique; (4) Outcomes: studies reporting preoperative and postoperative ASA positivity rates or ASA levels; and (5) Availability: full-text articles were accessible.

### 2.3 Exclusion criteria

Studies were excluded if they met any of the following criteria: (1) Publication type: conference abstracts, conference proceedings, case reports, case series with fewer than 10 participants, reviews, expert opinions, book chapters, animal studies, or *in vitro* studies; (2) Population: patients with subclinical varicocele, adolescent patients, or secondary varicocele; (3) Intervention: non-surgical management, including conservative medical therapy or assisted reproductive techniques; (4) Outcomes: insufficient or unavailable outcome data; or (5) Publication duplication: duplicate publications or studies with overlapping populations, in which case only the study providing the most complete data was included.

### 2.4 Literature screening and data extraction

Two reviewers independently screened the retrieved studies and extracted relevant data. Studies were initially screened based on titles and abstracts, followed by full-text assessment to determine eligibility. Any disagreements were resolved through discussion, and a third reviewer was consulted when consensus could not be reached.

The following data were extracted from each included study: first author, publication year, country, study design, sample size, participant characteristics (including mean age, varicocele grade, and laterality), ASA positivity threshold, surgical technique, follow-up duration, preoperative and postoperative numbers of ASA-positive patients and total participants, and mean ± standard deviation (SD) of ASA levels when available. Information related to study quality assessment was also recorded.

For studies reporting ASA positivity rates without providing the corresponding number of positive cases, the number of ASA-positive patients was estimated by multiplying the reported rate by the total sample size and rounding to the nearest integer. All extracted data were entered into a standardized Excel spreadsheet and independently verified for accuracy.

### 2.5 Quality assessment

The methodological characteristics of the included studies were assessed using the Joanna Briggs Institute (JBI) Critical Appraisal Checklist for Case Series. The checklist consists of 10 items, each rated as “yes,” “no,” “unclear,” or “not applicable.” The overall assessment was based on the number of items rated as “yes”: studies with ≥8 positive responses were considered to have high methodological quality, those with 6–7 positive responses as moderate quality, and those with ≤5 positive responses as low quality. Because the JBI checklist is primarily designed to evaluate the methodological and reporting quality of case series, it does not fully assess issues such as confounding, selection bias, or causal inference limitations associated with uncontrolled before-after studies. Therefore, the quality assessment results were interpreted with consideration of these limitations. Two reviewers independently performed the quality assessment, and disagreements were resolved through discussion. The assessment results are presented in **Table 2**.

**Table 2:** Methodological quality assessment of included studies (JBI case series checklist).

Study	Q1	Q2	Q3	Q4	Q5	Q6	Q7	Q8	Q9	Q10	Total	Quality rating
Lin 2013	✓	✓	✓	×	✓	✓	✓	✓	✓	✓	9/10	High
Yu 2020	✓	✓	✓	✓	✓	✓	✓	✓	✓	✓	10/10	High
Yuan 2019	✓	✓	✓	✓	✓	✓	✓	✓	✓	✓	10/10	High
Luo 2016	✓	✓	✓	✓	✓	✓	✓	✓	✓	✓	10/10	High
Zhu 2018	✓	✓	✓	✓	✓	✓	✓	✓	✓	✓	10/10	High
Xue 2021	✓	✓	✓	✓	✓	✓	✓	✓	✓	✓	10/10	High
Zhang 2022	✓	✓	✓	✓	✓	✓	✓	✓	✓	✓	10/10	High
Zhong 2010	✓	✓	✓	?	✓	✓	?	✓	✓	✓	8/10	High
Liu 2023	✓	✓	✓	✓	✓	✓	✓	✓	✓	✓	10/10	High
Bozhedomov 2014	✓	✓	✓	✓	✓	✓	✓	✓	✓	✓	10/10	High

Q1–Q10 correspond to the JBI critical appraisal checklist for case series: Q1: clear inclusion criteria; Q2: standard measurement of condition; Q3: valid identification of condition; Q4: consecutive inclusion; Q5: complete inclusion; Q6: clear demographic information; Q7: clear clinical information; Q8: clear outcome/follow-up results; Q9: clear setting/location; Q10: appropriate statistical analysis.

√yes (criterion met); ?unclear (insufficient information). Quality rating: High (≥8 √), Moderate (6–7 √), Low (≤5 √).

### 2.6 Statistical analysis

Meta-analyses were performed using Review Manager (RevMan) version 5.4.1. For dichotomous outcomes, the risk ratio (RR) with 95% confidence intervals (CIs) was used to evaluate differences in ASA positivity rates. For continuous outcomes, the standardized mean difference (SMD) with 95% CIs was calculated for changes in ASA levels. Considering the expected clinical and methodological heterogeneity among studies, a random-effects model using the DerSimonian–Laird method was applied for all analyses. Statistical heterogeneity was assessed using Cochran’s Q test and the I² statistic. An I² value of <50% was considered low heterogeneity, 50–75% moderate heterogeneity, and >75% substantial heterogeneity. Subgroup analyses were performed according to surgical technique, including microsurgical, laparoscopic, and high-ligation approaches. Sensitivity analyses were conducted by excluding studies requiring estimation of ASA-positive cases from reported percentages and studies with different follow-up durations to evaluate the robustness of the pooled results. Publication bias was assessed using funnel plots when ≥10 studies were available. If funnel plot asymmetry was observed, Egger’s regression test was performed, with P < 0.05 considered statistically significant. All statistical tests were two-sided, and a P value <0.05 was considered statistically significant. Additional calculations were performed using Microsoft Excel when required.

## 3 Results

### 3.1 Literature search results

**Figure 1:**
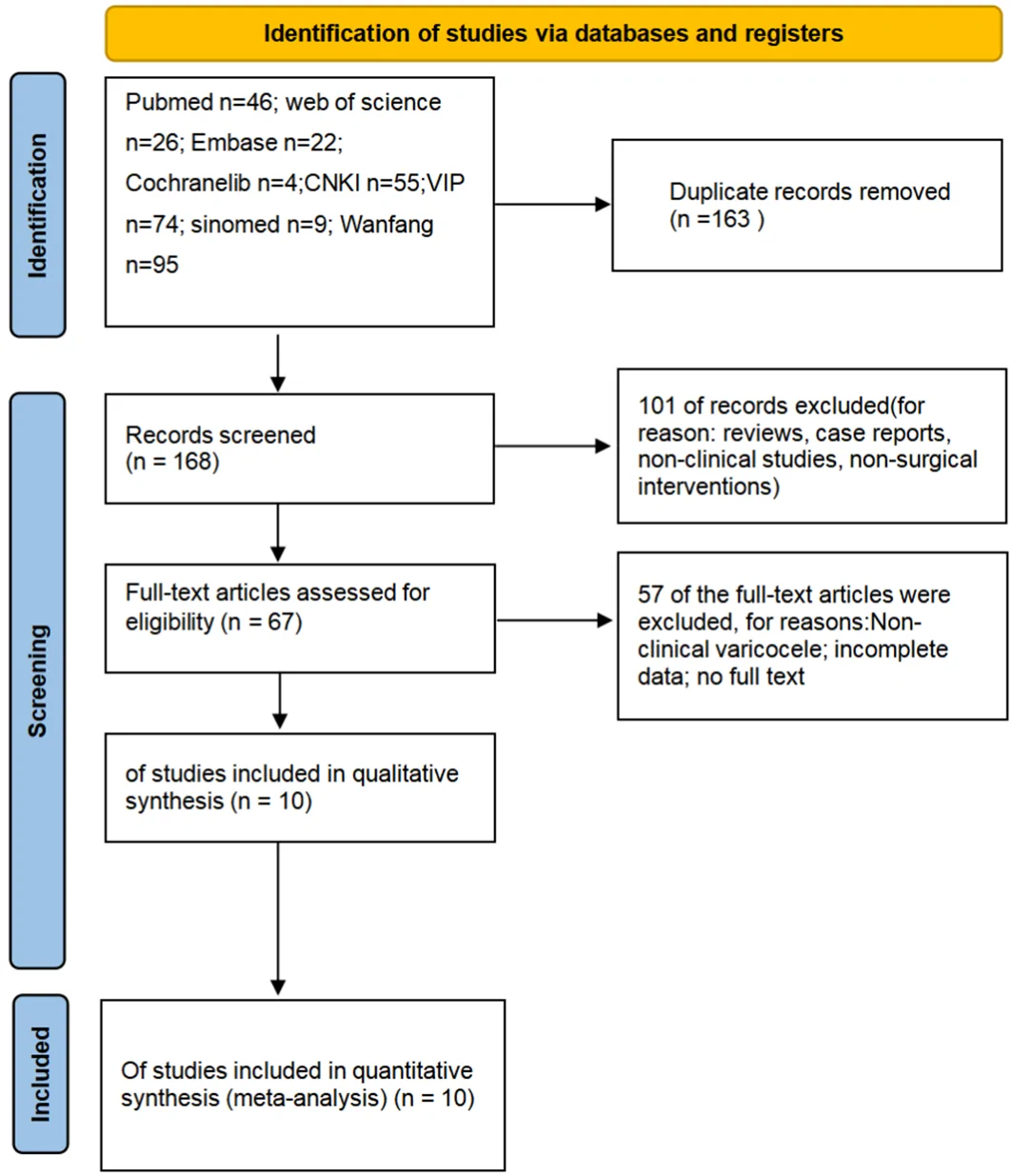
PRISMA flow diagram.

Notes: The Preferred Reporting Items for Systematic Reviews and Meta-Analyses (PRISMA).

The systematic search of PubMed, Web of Science, Embase, Cochrane Library, CNKI, VIP, Wanfang, and SinoMed identified 331 records. After removal of duplicates, 168 records remained for title and abstract screening. Of these, 101 records were excluded because they did not meet the eligibility criteria, including reviews, case reports, non-clinical studies, and studies involving non-surgical interventions. The full texts of the remaining 67 records were assessed for eligibility. Studies were excluded because they involved patients with subclinical or secondary varicocele, included adolescent populations, lacked preoperative or postoperative ASA data, or provided insufficient information for data extraction. Finally, 10 studies were included in the meta-analysis (Lin et al., 2013 (20); Yu et al., 2020 (21); Yuan et al., 2019 (22); Luo et al., 2016 (23); Zhu et al., 2018 (24); Xue et al., 2021 (25); Zhang et al., 2022 (26); Zhong et al., 2010 (27); Liu et al., 2023 (28); Bozhedomov et al., 2014 (15)). Among the included studies, nine were conducted in China and one in Russia, with a total of 830 participants. The included studies reported preoperative and postoperative ASA outcomes after varicocele repair, with a follow-up duration of approximately 3 months. The study selection process is illustrated in the PRISMA flow diagram (**Figure 1**).

### 3.2 Characteristics of the included studies

The characteristics of the included studies are summarized in **Table 3**.

**Table 3:** Characteristics of included studies.

Surgical approach	Study design	Sample size (pre/post)	Age (years, mean ± SD or range)	Varicocele grade (n)	Follow-up (months)	ASA detection method	Data type	
Lin 2013	Laparoscopic	Case series	80/80	22–37	I:17, II:43, III:20	3	ELISA (seminal IgA)	Dichotomous
Yu 2020 (microsurgery)	Microsurgical	Case series	44/44	28.85 ± 5.65 (21–38)	II:25, III:19	3	ELISA (serum)	Dichotomous
Yu 2020 (high ligation)	High ligation	Case series	44/44	28.63 ± 5.41 (21–36)	II:27, III:17	3	ELISA (serum)	Dichotomous
Yuan 2019	Microsurgical	Case series	67/67	28.15 ± 6.08 (19–38)	II/III (exact not reported)	6 (3-month data: 29/67, P > 0.05, not used)	ELISA (serum + seminal)	Dichotomous
Luo 2016 (microsurgery)	Microsurgical	Case series	67/67	28.7 ± 6.0 (22–39)	II:40, III:26	3	ELISA (serum)	Dichotomous
Luo 2016 (conventional)	High ligation	Case series	53/53	29.0 ± 5.8 (22–37)	II:33, III:20	3	ELISA (serum)	Dichotomous
Zhu 2018 (laparoscopic) (Data were back-calculated from reported percentages and total sample sizes.)	Laparoscopic	Case series	48/48	26.37 ± 5.86	II:20, III:28	3	ELISA (serum)	Dichotomous
Zhu 2018 (microsurgery)	Microsurgical	Case series	53/53	26.84 ± 6.24	II:31, III:22	3	ELISA (serum)	Dichotomous
Xue 2021 (laparoscopic)	Laparoscopic	Case series	61/61	28.74 ± 5.26 (21–36)	II:35, III:26	3	ELISA (serum)	Dichotomous
Xue 2021 (microsurgery)	Microsurgical	Case series	61/61	29.01 ± 5.87 (22–38)	II:34, III:27	3	ELISA (serum)	Dichotomous
Zhang 2022 (microsurgery)	Microsurgical	Case series (RCT design)	48/48	27.56 ± 3.52	II:23, III:25	3	ELISA (serum)	Dichotomous
Zhang 2022 (laparoscopic)	Laparoscopic	Case series (RCT design)	48/48	27.62 ± 3.48	II:24, III:24	3	ELISA (serum)	Dichotomous
Zhang 2022 (high ligation)	High ligation	Case series (RCT design)	48/48	27.59 ± 3.55	II:26, III:22	3	ELISA (serum)	Dichotomous
Zhong 2010	High ligation	Case series	35/35	25–40 (mean 33)	NR	3–6	MAR (seminal)	Continuous
Liu 2023	Laparoscopic	Case series	40/40	32.12 ± 5.24 (18–46)	I:9, II:22, III:9	3	MAR (seminal)	Continuous
Bozhedomov 2014	Microsurgical	Prospective cohort	33/33	31–33 (range)	Clinical grade not specified	3	MAR (seminal IgG)	Continuous

Antisperm antibody(ASA), Enzyme-linked immunosorbent assay(ELISA), mixed antiglobulin reaction(MAR), Continuous data were not pooled due to high heterogeneity (I² = 98%) and are described narratively.

### 3.3 Quality of included literature

According to the JBI Case Series Checklist, all 10 included studies were classified as having high methodological quality. However, this rating reflects adherence to case-series assessment criteria and does not necessarily indicate high certainty of evidence, particularly given the potential influence of confounding factors and limitations in causal inference. The quality assessment results are shown in **Table 2**.

### 3.4 Effect of varicocele repair on ASA positivity rate

**Figure 2:**
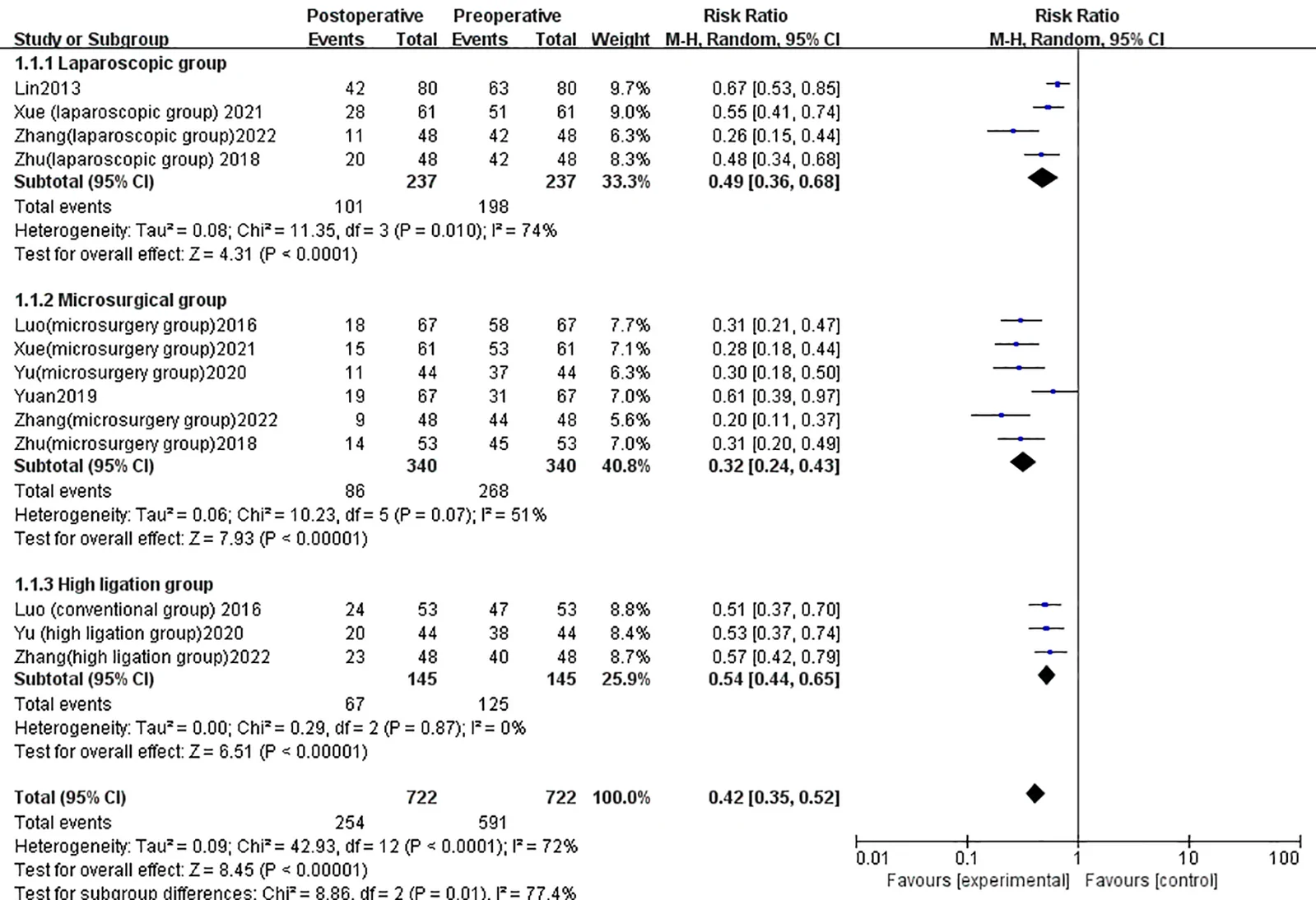
Forest plot comparing the pre- and post-operative ASA positivity.

A total of 13 comparisons from 10 studies, including 722 participants, were included in the analysis of ASA positivity rates. Compared with baseline preoperative values, ASA positivity rates were lower after varicocele repair (RR = 0.42, 95% CI: 0.35–0.52, *P* < 0.00001; **Figure 2**). Significant heterogeneity was observed among the included studies (I² = 72%, *P* < 0.0001).

Subgroup analyses were conducted according to surgical technique. In the laparoscopic subgroup (4 comparisons, n = 237), the pooled RR was 0.49 (95% CI: 0.36–0.68; I² = 74%, *P* < 0.0001). In the microsurgical subgroup (6 comparisons, n = 340), the pooled RR was 0.32 (95% CI: 0.24–0.43; I² = 51%, *P* = 0.07). In the high-ligation subgroup (3 comparisons, n = 145), the pooled RR was 0.54 (95% CI: 0.44–0.65; I² = 0%). The test for subgroup differences showed a statistically significant difference among surgical techniques (χ² = 8.86, df = 2, *P* = 0.01; I² = 77.4%). Among the evaluated surgical approaches, the microsurgical subgroup had the lowest pooled RR, whereas the high-ligation subgroup showed the highest pooled RR.

Notes: Confidence interval (CI), standard deviation (SD).

### 3.5 Heterogeneity and subgroup analysis

Substantial heterogeneity was observed in the overall analysis (I² = 72%, *P* < 0.0001). This heterogeneity may be related to differences in clinical characteristics and study methodologies among the included studies. Subgroup analyses based on surgical technique were performed to explore potential sources of variability.

Heterogeneity was low in the high-ligation subgroup (I² = 0%), whereas moderate-to-substantial heterogeneity remained in the laparoscopic (I² = 74%) and microsurgical (I² = 51%) subgroups. These findings suggest that surgical technique may explain part, but not all, of the observed heterogeneity. Other potential contributing factors include differences in baseline ASA positivity, varicocele grade, infertility characteristics, ASA detection methods, positivity thresholds, follow-up duration, and surgical procedures across centers. Because most included studies did not provide individual-level data, further analyses using adjusted models or meta-regression were not feasible. Nevertheless, the direction of the pooled effects was consistent across all surgical subgroups (all RRs < 1), indicating that lower ASA positivity after varicocele repair was consistently observed despite the presence of heterogeneity.

### 3.6 Sensitivity analysis

**Figure 3:**
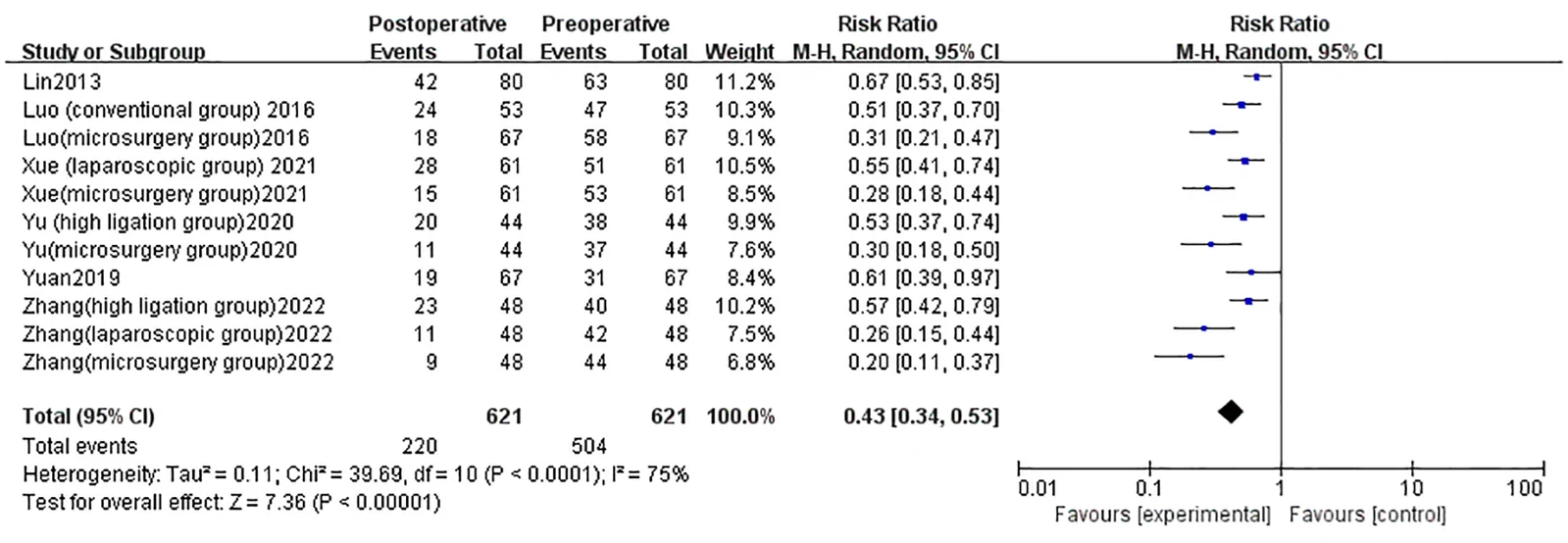
Sensitivity analysis forest plot for ASA positivity rate after excluding the study with back-calculated data.

**Figure 4:**
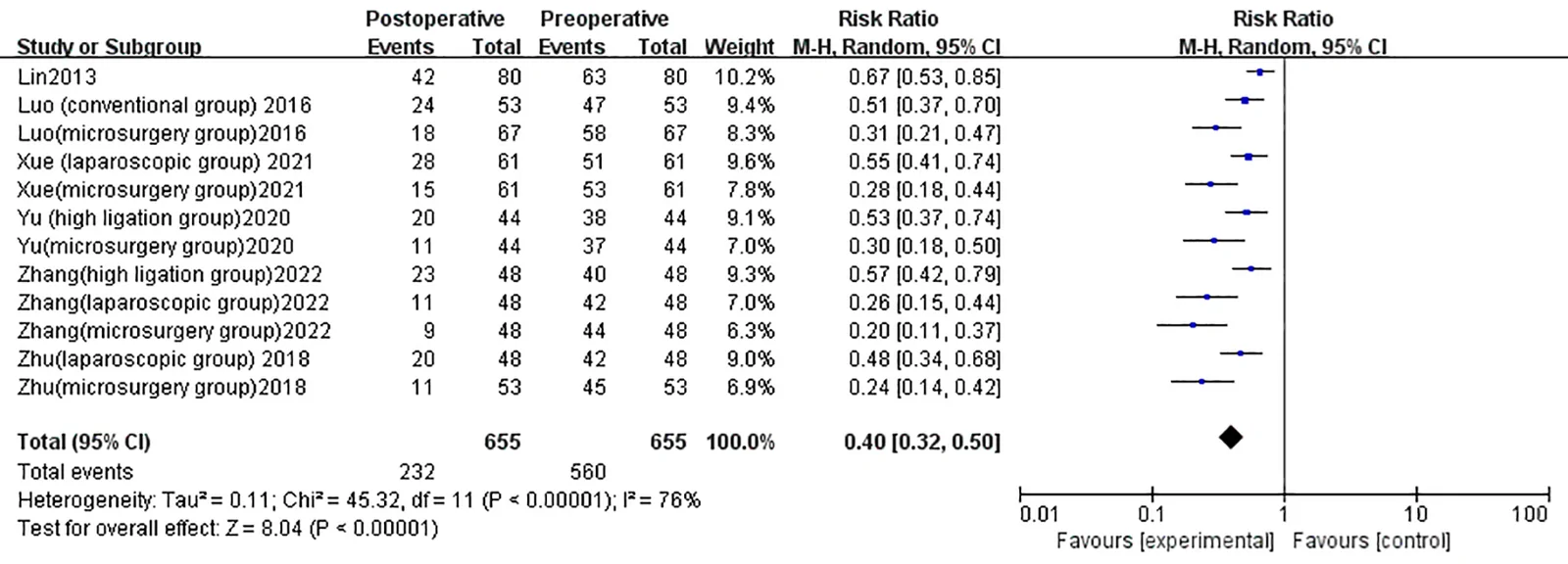
Sensitivity analysis forest plot for ASA positivity rate after excluding the 6-month follow-up study.

Two sensitivity analyses were performed to evaluate the robustness of the primary findings. First, the study with estimated ASA-positive cases derived from reported percentages (Zhu et al., 2018 (24))was excluded. The pooled RR remained similar to the primary analysis (RR = 0.43, 95% CI: 0.34–0.53, I² = 75%; **Figure 3** vs. RR = 0.42, 95% CI: 0.35–0.52). Second, we excluded the study with a 6-month follow-up (Yuan et al., 2019 (22)). was excluded. The pooled RR was 0.40 (95% CI: 0.32–0.50, I² = 76%; **Figure 4**), which was also comparable with the primary estimate. Overall, the sensitivity analyses indicated that the pooled results were not substantially influenced by the inclusion of studies with estimated data or different follow-up durations.

### 3.7 Continuous outcomes: ASA levels

**Figure 5:**
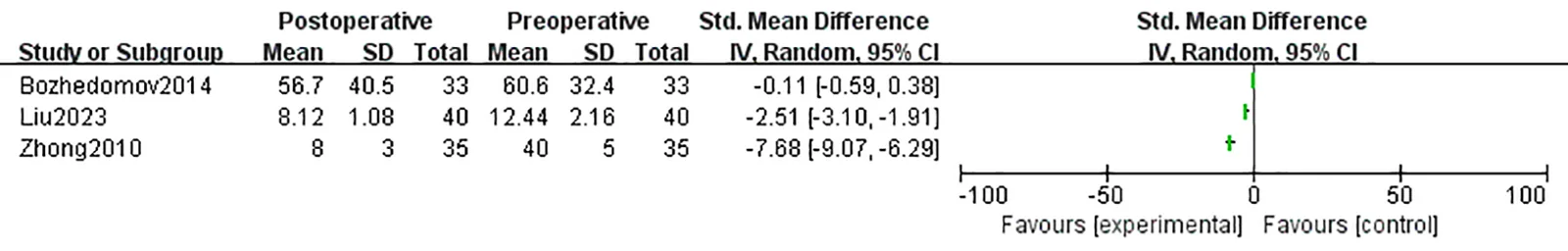
Forest plot of individual study results for antisperm antibody levels (continuous data).

Three studies reported ASA levels as continuous outcomes (mean ± standard deviation). Due to substantial heterogeneity among these studies (I² = 98%, *P* < 0.00001; **Figure 5**), quantitative pooling was not performed, and the findings were summarized descriptively.

As shown in **Figure 5**, Zhong et al. (27)reported a decrease in ASA levels from 40.0% ± 5.0% before surgery to 8.0% ± 3.0% after surgery (n = 35; SMD = -7.68, 95% CI: -9.07 to -6.29). Liu et al. (28)also observed a decrease in ASA levels, from 12.44% ± 2.19% preoperatively to 8.12% ± 1.08% postoperatively (n = 40; SMD = −2.51, 95% CI: −3.10 to −1.91). In contrast, Bozhedomov et al (15)reported no significant change, with ASA levels remaining similar before and after surgery (60.6% ± 32.4% vs. 56.7% ± 40.5%; n = 33; SMD = −0.11, 95% CI: −0.59 to 0.38). The high heterogeneity observed among studies reporting continuous ASA levels may be related to differences in ASA detection methods (MAR versus ELISA), antibody targets, baseline ASA concentrations, patient characteristics, surgical techniques, and follow-up duration. Therefore, these findings were interpreted descriptively and should be considered with caution. The primary conclusions of this meta-analysis were based on the dichotomous outcome of ASA positivity rate.

### 3.8 Publication bias assessment

**Figure 6:**
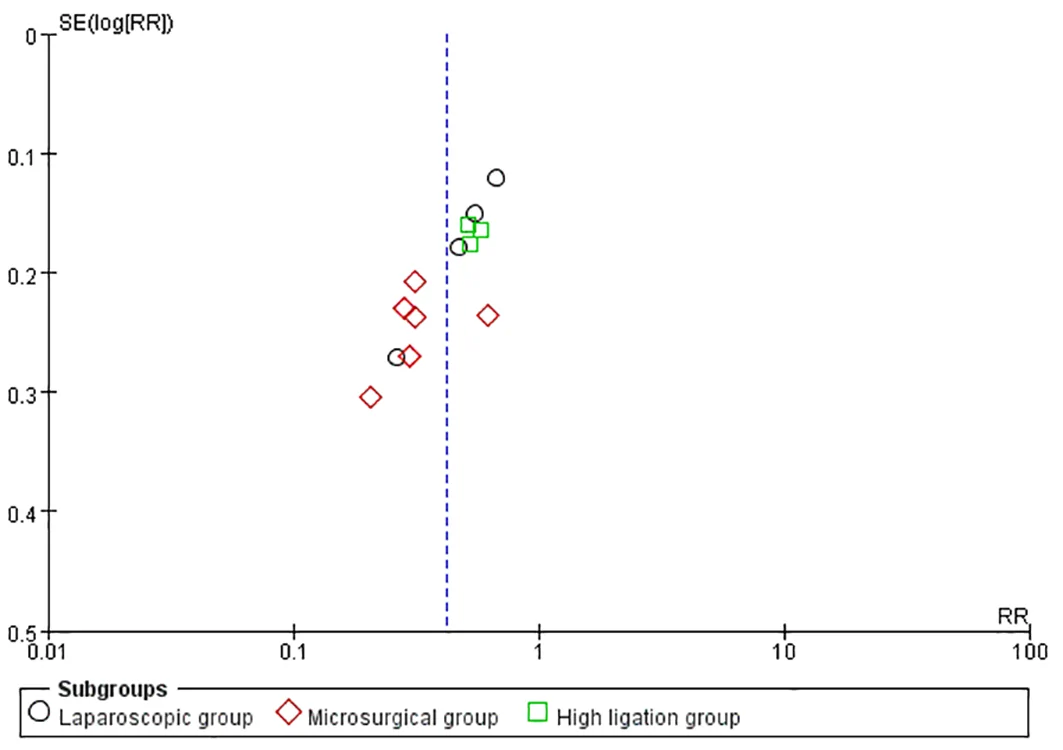
Funnel plot for publication bias assessment of the primary meta-analysis (13 comparisons).

A funnel plot was generated to assess potential publication bias in the primary meta-analysis (**Figure 6**). Visual inspection showed no apparent asymmetry, with study estimates distributed approximately symmetrically around the pooled effect estimate (RR = 0.42). The limited number of studies with effects in the opposite direction may reflect the overall consistency of the observed association rather than clear evidence of publication bias. Overall, no apparent evidence of publication bias was identified based on funnel plot assessment.

## 4 Discussion

This meta-analysis provides the first quantitative synthesis evaluating changes in ASA positivity after varicocele repair. The pooled analysis of 10 studies demonstrated a lower ASA positivity rate after varicocele repair compared with preoperative values (RR = 0.42, 95% CI: 0.35–0.52). Subgroup analysis by surgical technique revealed that microsurgical varicocele repair showed the lowest pooled (RR = 0.32), followed by laparoscopic surgery (RR = 0.49) and high ligation (RR = 0.54). These findings help address an important gap in the current literature, as previous meta-analyses of varicocele repair have primarily focused on conventional semen parameters, sperm DNA fragmentation, or fertility-related outcomes. By systematically evaluating changes in ASA positivity and exploring differences among surgical approaches, this study provides additional insights into the potential immunological alterations associated with varicocele repair.

Substantial heterogeneity was observed in the overall analysis (I² = 72%, *P* < 0.0001). Such heterogeneity is common in meta-analyses of surgical interventions and may result from differences in patient characteristics, surgical techniques, follow-up duration, and outcome assessment methods. In this study, subgroup and sensitivity analyses were performed to explore potential sources of variability and evaluate the robustness of the findings. Subgroup analysis according to surgical technique suggested that the type of surgical approach may partly contribute to the observed heterogeneity. The high-ligation subgroup showed relatively consistent results with low heterogeneity, whereas greater variability was observed in the laparoscopic and microsurgical subgroups. These differences may reflect variations in operative procedures, surgeon experience, patient characteristics, or baseline disease severity among studies. Nevertheless, the direction of the association was consistent across all subgroups (all RRs < 1), and the test for subgroup differences was statistically significant (P = 0.01), suggesting that surgical technique may be an important factor associated with differences in postoperative ASA positivity outcomes, suggesting that surgical technique may influence the observed association between varicocele repair and changes in ASA positivity. Sensitivity analyses further supported the stability of the findings. Excluding the study with a 6-month follow-up (Yuan et al., 2019 (22)) or the study requiring estimation of ASA-positive cases from reported percentages (Zhu et al.,2018 (24)) resulted in pooled RRs ranging from 0.40 to 0.43, which were comparable with the primary estimate (RR = 0.42). These results suggest that the overall findings were not substantially affected by individual studies or specific methodological variations. Therefore, although heterogeneity remained among the included studies, the consistency of the observed association across subgroup and sensitivity analyses supports the robustness of the main findings. However, these results should be interpreted in the context of the limitations inherent to the available study designs.

Evidence from VC animal models supports an association between disruption of the BTB and the formation of ASA (29). Pan et al. (30)and Raitsina et al. (31)both reported significant disruption of the BTB in VC models, with the latter also finding that lymphocytes in lymphatic organs became sensitized to sperm antigens. Soares et al. (32)proposed that VC-induced upregulation of the receptor for activated C kinase 1 (RACK1) may be involved in the phosphorylation of focal adhesion kinase (FAK), thereby affecting the dynamic stability and external structure of the BTB. Immune factors in VC act on tight junction molecules of the BTB, increasing its permeability (29); consequently, immune factors enter the seminiferous tubules and destroy spermatogenic cells, while sperm antigens become exposed to the circulation. Tight junction proteins involved in cell adhesion and BTB formation-such as claudin-11 (expressed by Sertoli cells), E-cadherin, and α-catenin-are significantly downregulated in the seminiferous tubules (33–35). These changes compromise BTB integrity, facilitate sperm antigen exposure, and ultimately lead to ASA production. Based on these mechanisms, varicocele repair may be associated with changes in ASA production, potentially through improvement of the testicular microenvironment and restoration of blood-testis barrier function. The observed reduction in ASA positivity after varicocele repair in our meta-analysis (RR = 0.42, 95% CI: 0.35–0.52) is consistent with an association between varicocele repair and immune-related alterations. However, whether these changes represent a direct causal effect of surgery remains uncertain and requires confirmation in well-designed prospective studies.

Although our meta-analysis demonstrated a statistically significant overall reduction in ASA positivity after varicocele repair, these findings require careful interpretation considering the complex relationship between varicocele, ASA, and male infertility. First, ASA positivity is not a universal feature of clinical varicocele. In the general population, ASA positivity is uncommon, with reported prevalence generally ranging from 0% to 2% (36). Among infertile men without clinical varicocele, ASA positivity appears to be higher but remains relatively limited, with reported rates of approximately 5%–15% (36). In contrast, infertile men with varicocele have been reported to exhibit higher ASA positivity rates; however, a substantial proportion of patients with varicocele remain ASA-negative, and ASA positivity can also occur in infertile men without varicocele. In the included studies, baseline ASA positivity rates varied considerably, and many patients had negative ASA results before surgery. This heterogeneity suggests that ASA elevation does not represent a consistent feature of varicocele-related testicular dysfunction. Importantly, the absence of ASA positivity should not be interpreted as evidence that varicocele repair would be ineffective, as the decision to perform surgery is based on comprehensive clinical factors, including varicocele characteristics, semen abnormalities, and reproductive considerations, rather than ASA status alone. Therefore, ASA should be considered as one potential immune-related mechanism involved in varicocele-associated infertility rather than a universal or specific marker of the disease.

In addition, although the pooled analysis of dichotomous ASA outcomes indicated a reduction in ASA positivity after surgery, this finding was not consistently observed across all included studies. For instance, Bonyadi et al. (16)reported no significant changes in ASA levels following varicocele ligation. Such discrepancies may reflect differences in surgical techniques, the extent of tissue manipulation, postoperative assessment timing, and individual immune responses. These factors suggest that surgical intervention may influence ASA levels through complex and variable mechanisms. Therefore, the reduction in ASA positivity identified in our meta-analysis should be interpreted primarily as evidence of changes in ASA status after varicocele repair, rather than direct evidence of improved immune function or enhanced fertility outcomes. Further studies combining ASA assessment with semen parameters, pregnancy outcomes, and long-term follow-up are required to determine whether postoperative changes in ASA status have meaningful clinical implications.

Another important consideration is the geographic distribution of the available evidence. All studies reporting a significant reduction in ASA positivity after varicocele repair were conducted in China, whereas the only non-Chinese study included in our meta-analysis, by Bozhedomov et al. (15), found no significant change after surgery. Similarly, other studies from non-Chinese populations have reported stable or even increased postoperative ASA positivity (16, 17). This geographic imbalance limits the generalizability of our findings. Differences in genetic background, lifestyle, body composition, baseline semen characteristics, infertility profiles, healthcare-seeking behaviors, and clinical management strategies may influence both baseline ASA prevalence and postoperative changes. Moreover, although microsurgical varicocele repair is widely performed, variations in surgical techniques, including the extent of venous ligation, preservation of arteries and lymphatic vessels, and surgeon experience, may also contribute to differences in reported outcomes.

These geographic differences are further reflected in the baseline ASA characteristics of the included studies. The Chinese cohorts in our meta-analysis generally reported relatively high baseline ASA positivity among patients with VC, whereas previous international systematic reviews and meta-analyses have reported pooled ASA positivity rates of approximately 39.8% by MAR testing, 50% by immunobead testing, and 37.1% by ELISA in men with VC (37). However, direct comparisons between populations remain challenging because differences in diagnostic methods, cutoff values, patient selection, clinical characteristics, and study design may substantially affect reported ASA rates. Therefore, the observed reduction in ASA positivity after varicocele repair should be interpreted within the context of these geographic and methodological variations. Future studies involving diverse populations and standardized assessment protocols are needed to determine whether similar changes in ASA status occur across different clinical settings.

Several limitations of this meta-analysis should be considered. First, the available evidence was geographically limited, as most included studies were conducted in China, with only minimal data from other populations. Therefore, the generalizability of our findings remains uncertain. Differences in patient characteristics, infertility profiles, healthcare practices, and surgical management across regions may influence both baseline ASA status and postoperative changes.

Second, all included studies used before–after designs without concurrent untreated control groups. Although a reduction in ASA positivity was observed after varicocele repair, this change cannot be definitively attributed to surgery alone. Spontaneous fluctuations in antibody levels, regression to the mean, and the natural course of infertility may also contribute to changes in ASA status. Third, although ASA positivity may be more common among men with varicocele than in the general population, ASA elevation is not a universal feature of varicocele and can also occur in infertile men without varicocele. Therefore, ASA likely represents only one potential mechanism involved in varicocele-associated infertility, and the clinical relevance of changes in ASA status after repair remains unclear. Moreover, the included studies did not report important reproductive outcomes, such as pregnancy rates, live birth rates, or assisted reproductive outcomes. Consequently, whether postoperative changes in ASA status translate into improved fertility outcomes remains unknown. Fourth, although all included studies were rated as “high quality” according to the JBI Case Series Checklist, this tool is primarily designed to evaluate methodological characteristics and reporting quality of case series rather than confounding control or causal inference. Therefore, the overall certainty of evidence should be interpreted cautiously given the uncontrolled nature of the included studies. Fifth, substantial heterogeneity was observed in continuous ASA level outcomes (I² = 98%), and the included studies reported inconsistent findings. For this reason, quantitative pooling of ASA levels was not considered appropriate, and these data were summarized descriptively. Variations in ASA detection methods, baseline antibody levels, patient characteristics, and surgical techniques may have contributed to this heterogeneity. Finally, the follow-up periods of the included studies were relatively short. Most studies assessed outcomes at approximately 3 months after surgery, and only one study reported 6-month follow-up data. Therefore, the durability of postoperative ASA changes remains uncertain. Future prospective studies with longer follow-up, appropriate control groups, standardized ASA assessment methods, and clinically relevant reproductive outcomes are needed to clarify the long-term significance of ASA changes after varicocele repair.

Future studies should focus on determining whether changes in ASA status after varicocele repair have meaningful clinical implications. Pregnancy and other fertility-related outcomes should be included as primary endpoints to clarify whether reduced ASA positivity is associated with improved reproductive success. In addition, standardized ASA detection methods and consistent definitions of positivity are needed to minimize heterogeneity and improve comparability across studies. Long-term follow-up, preferably extending beyond 12 months, is also required to determine whether postoperative changes in ASA status are sustained and whether they are associated with persistent improvements in semen quality. Furthermore, randomized controlled trials comparing microsurgical repair with other surgical approaches, particularly among patients with ASA positivity, would provide stronger evidence regarding the most appropriate surgical strategy for this subgroup.

Given the current geographic concentration of available evidence, international multicenter studies involving diverse populations and standardized protocols are essential to validate the generalizability of these findings and better define the role of ASA changes after varicocele repair.

## 5 Conclusion

This meta-analysis suggests that varicocele repair is associated with a reduction in ASA positivity among men with clinical VC, with microsurgical repair showing a greater observed decrease compared with other approaches. However, these findings are based on uncontrolled before–after studies and should be interpreted cautiously. The substantial heterogeneity among studies, limited evidence from non-Chinese populations, and lack of data demonstrating a link between postoperative ASA changes and improved fertility outcomes limit the clinical implications of these findings. Therefore, ASA testing alone should not currently be used to guide surgical decision-making. Future randomized controlled trials with long-term follow-up and clinically relevant fertility outcomes are needed to clarify the significance of ASA changes after varicocele repair.
