# Supplementary material for: Effect of varicocele surgical repair on antisperm antibody positivity rate in patients with clinical varicocele: a systematic review and meta-analysis
Source: Front Endocrinol (Lausanne). 2026 Aug 12;17:1925219. doi: 10.3389/fendo.2026.1925219 (PMC13506440; doi:10.3389/fendo.2026.1925219)
Supplement: Supplementary file 2 [file Table2.docx]

Supplementary Methods: Search Strategy

Search period: From database inception to April 30, 2026

Web of Science

| # | Search |
| --- | --- |
| #1 | TS=("varicocele" OR "varicoceles" OR "varicocelectomy" OR "internal spermatic vein") |
| #2 | TS=("varicocelectomy" OR "varicocele repair" OR "surgical repair" OR "high ligation" OR "microsurgical varicocelectomy" OR "laparoscopic varicocelectomy" OR "varicocele ligation") |
| #3 | TS=("antisperm antibodies" OR "antisperm antibody" OR "anti-sperm antibody" OR "ASA" OR "sperm antibody" OR "sperm antibodies" OR "sperm agglutinating antibody" OR "sperm surface antibody" OR "immunological infertility") |
| #4 | #1 AND #2 AND #3 |

EMbase

| # | Search |
| --- | --- |
| #1 | exp varicocele/ |
| #2 | (varicocele or varicoceles or varicocelectomy or internal spermatic vein).tw. |
| #3 | or/1-2 |
| #4 | exp varicocelectomy/ |
| #5 | (varicocelectomy or varicocele repair or surgical repair or high ligation or microsurgical varicocelectomy or laparoscopic varicocelectomy or varicocele ligation).tw. |
| #6 | or/4-5 |
| #7 | exp antisperm antibody/ |
| #8 | (antisperm antibodies or antisperm antibody or anti-sperm antibody or ASA or sperm antibody or sperm antibodies or sperm agglutinating antibody or sperm surface antibody or immunological infertility).tw. |
| #9 | or/7-8 |
| #10 | (before-after or pre-post or preoperatively or postoperatively or prospective or retrospective).tw. |
| #11 | #3 and #6 and #9 and #10 |

Cochrane Library

| # | Search |
| --- | --- |
| #1 | MeSH descriptor: [Varicocele] explode all trees |
| #2 | (varicocele or varicoceles or varicocelectomy or "internal spermatic vein"):ti,ab,kw |
| #3 | #1 or #2 |
| #4 | MeSH descriptor: [Varicocelectomy] explode all trees |
| #5 | (varicocelectomy or "varicocele repair" or "surgical repair" or "high ligation" or "microsurgical varicocelectomy" or "laparoscopic varicocelectomy"):ti,ab,kw |
| #6 | #4 or #5 |
| #7 | MeSH descriptor: [Antisperm Antibodies] explode all trees |
| #8 | (antisperm antibodies or antisperm antibody or anti-sperm antibody or ASA or sperm antibody or sperm antibodies or sperm agglutinating antibody or sperm surface antibody or immunological infertility):ti,ab,kw |
| #9 | #7 or #8 |
| #10 | #3 and #6 and #9 |

China National Knowledge Infrastructure (CNKI)

(TKA='精索静脉曲张' OR TKA='精索静脉曲张术' OR TKA='精索静脉高位结扎术' OR TKA='显微镜下精索静脉结扎术' OR TKA='腹腔镜精索静脉结扎术' OR TKA='精索静脉结扎' OR TKA='精索内静脉高位结扎术') AND (TKA='抗精子抗体' OR TKA='抗精抗体' OR TKA='ASA' OR TKA='AsAb' OR TKA='精子抗体' OR TKA='精子表面抗体' OR TKA='免疫性不育') AND (TKA='术前' OR TKA='术后' OR TKA='手术前后')

Filter by: synonym expansion

China Science and Technology Journal Database (VIP)

(M=(精索静脉曲张 OR 精索静脉曲张术 OR 精索静脉高位结扎术 OR 显微镜下精索静脉结扎术 OR 腹腔镜精索静脉结扎术 OR 精索静脉结扎 OR 精索内静脉高位结扎术)) AND (M=(抗精子抗体 OR 抗精抗体 OR ASA OR AsAb OR 精子抗体 OR 精子表面抗体 OR 免疫性不育)) AND (R=(术前 OR 术后 OR 手术前后))

Chinese Biomedical Literature Database (SinoMed)

(('精索静脉曲张' OR '精索静脉曲张术' OR '精索静脉高位结扎术' OR '显微镜下精索静脉结扎术' OR '腹腔镜精索静脉结扎术' OR '精索静脉结扎' OR '精索内静脉高位结扎术') 常用字段:智能) AND (('抗精子抗体' OR '抗精抗体' OR 'ASA' OR 'AsAb' OR '精子抗体' OR '精子表面抗体' OR '免疫性不育') 常用字段:智能) AND (('术前' OR '术后' OR '手术前后') 常用字段:智能)

Wanfang Data

(题名或关键词:(精索静脉曲张 OR 精索静脉曲张术 OR 精索静脉高位结扎术 OR 显微镜下精索静脉结扎术 OR 腹腔镜精索静脉结扎术 OR 精索静脉结扎 OR 精索内静脉高位结扎术)) AND (题名或关键词:(抗精子抗体 OR 抗精抗体 OR ASA OR AsAb OR 精子抗体 OR 精子表面抗体 OR 免疫性不育)) AND (摘要:(术前 OR 术后 OR 手术前后))
